# Behavioural Change in Practice: Primary Care Providers’ Journey Towards Goal-Oriented Care

**DOI:** 10.5334/ijic.9067

**Published:** 2025-12-19

**Authors:** Reini Haverals, Sibyl Anthierens, Peter Pype, Carolyn Steele Gray, Kris Van den Broeck, Pauline Boeckxstaens

**Affiliations:** 1 Department of Public Health and Primary Care, Faculty of Medicine and Health Sciences, Ghent University, Ghent, Belgium; 2 Department of Family Medicine and Population Health, Faculty of Medicine and Health sciences, University of Antwerp, Antwerp, Belgium; 3 Science of Care Institute, Lunenfeld-Tanenbaum Research Institute, Sinai Health, Toronto, Ontario, Canada; 4 Institute of Health Policy, Management & Evaluation, University of Toronto, Toronto, Ontario, Canada

**Keywords:** goal-oriented care, team-based primary care, person-centred integrated care, behaviour change, implementation

## Abstract

**Introduction:**

The demand for person-centred integrated care (PC-IC) requires health services focused on patients’ individual needs. Strengthening primary care is crucial in promoting PC-IC. Goal-oriented care (GOC) prioritizes patient goals and fosters interprofessional team-based care, optimizing PC-IC. GOC requires healthcare providers to shift from problem- to goal-oriented practices. However, how providers change their daily practice to align care with what matters most to patients remains unclear.

**Aim:**

This qualitative study explores how primary care providers (PCPs) experience behaviour change when implementing GOC in daily work after an interprofessional GOC-training.

**Method:**

Six months post-training, focus groups with PCPs were organized. A theoretical thematic analysis was conducted using the Capability, Opportunity, Motivation, and Behaviour (COM-B) model.

**Results:**

Twenty-two PCPs participated in five focus groups. Motivational factors catalysed behaviour change towards GOC, including developing awareness on care actions through reflective practice. PCPs identified capabilities such as asking person-centred questions, maintaining a broad knowledge and enhancing their advocacy for patients. Opportunities stressed team support, care continuity, and reflexivity-promoting workplaces as vital for enabling behavioural change in GOC.

**Conclusion:**

Reflective practice is vital for aligning PCPs’ behaviour with GOC. Involvement of all colleagues and dedicated time for reflection promote team alignment and consistency in achieving patients’ personal goals.

## Introduction

Worldwide, health systems are facing a growing demand for care and support for individuals with complex and long-term care needs. This demand has prompted health care systems to shift towards a person-centred integrated care approach, moving away from disease or problem-oriented models of care [1,2,3]. The World Health Organization defines person-centred integrated care (PC-IC) as an approach that addresses the needs and preferences of individuals, families, and communities by organizing integrated health services to provide a continuum of care at various levels and sites within the health system [1]. Strengthening primary care systems is seen as a key element in the global strategy to promote PC-IC. Both research and policy emphasize the significant role of primary care in coordinating a patient’s healthcare journey [1,4].

A growing concept in literature with a person-centred perspective on healthcare organization, is goal-oriented care (GOC) [5]. In a GOC approach, patient’s personal goals and preferences serve as a compass for organizing care and support, consistently shaped by their context and values [6]. In other words, GOC means making a shift from ‘*what is the matter with the patient*’ to ‘*what matters most to the patient*’. Recognizing the dynamic nature of goals, GOC emphasizes an iterative process, necessitating regular evaluations to ensure that care remains aligned with patients’ goals [5]. At the patient-provider level, GOC offers a practical framework for aligning care plans with patients’ personal goals rather than objectives tied to specific conditions, providers, or organizational workflows. By using the patient’s goals as a guiding principle, GOC fosters a person-focused approach that encourages meaningful dialogue, mutual understanding, and care decisions centred on what truly matters to patients [7,8]. At the team level, GOC provides a unifying philosophy for primary care providers (PCPs) by creating shared goals rooted in the patient’s priorities. This shared focus bridges professional boundaries, enabling teams to coordinate roles and responsibilities more effectively [9]. By fostering interconnectedness, GOC enhances interprofessional collaboration and contributes to more cohesive care planning. While GOC can serve as a catalyst for person-centred integrated care, its potential to reduce fragmentation depends on alignment with supportive structures and policies at macro-, meso- and micro-levels [7,10].

Although the concept of GOC is highly recognized by PCPs as valuable for their work [11], it requires a shift in routines and strategies in their daily practices [12,13]. An observational study using video recordings among general practitioners (GPs) shows that even when GPs are encouraged to ask patients about personal goals, this information is not incorporated into care plans or actions [14]. These findings align with the experiences of chronic patients in primary care, who report that although their team of PCPs is open to discussing non-medical personal information, they do not experience this information is considered in care planning decisions [15]. Patients also observe that their providers often focus more on health-related goals rather than personal ones [16,17]. Therefore, the ability of GOC to catalyse person-centred integrated care depends on translating its theoretical potential into providers’ specific work contexts. Its implementation can reveal systemic barriers—such as payment systems, evaluation methods or local policies—and identify areas to strengthen the broader integrated care model [1,7]. As such, GOC can provide a practical framework for refining integrated care into a context-specific, actionable approach [18].

Ingraining GOC principles into the work and ethics of healthcare teams required behaviour change and new routines—understood as everyday practices that embed and sustain GOC [19]. Yet, there is limited evidence on how PCPs enact these changes over time, particularly regarding how their behaviour changes in daily practice as they align care with what matters most to patients [20]. This study addresses that gap by examining the behaviour change process as experienced by PCPs. While not the primary aim, understanding this process may also inform future workforce development by highlighting where education and organizational support can best enable change [21].

### The case of Flanders

The drive towards more PC-IC resonates in Flanders, Belgium’s Dutch-speaking region. In 2017, a primary care reform aimed to strengthen integrated care by creating networks across health and social care sectors, centred on individuals with care and support needs. This reform introduced 60 geographically defined primary care zones (PCZs) in Flanders and Brussels, each serving 75,000–125,000 residents with tailored services. Supported by the Flemish Institute for Primary Care (VIVEL), these zones aim to foster PC-IC locally by developing care teams that support individuals “in accordance with their life goals” [22]. To encourage providers to adopt this approach, VIVEL introduced goal-oriented care (GOC) as a guiding philosophy [23]. Research on GOC adoption in Flemish primary care shows that PCPs are highly motivated but uncertain about how to shift from problem-oriented to goal-oriented care. Effective clinical integration requires both training in GOC and opportunities to apply it in practice [7]. However, a survey of 131 PCPs highlights a lack of training opportunities for GOC [11]. To address this, VIVEL developed an interprofessional pilot training on GOC, aligned with the integrated care vision [23]. Without existing literature on GOC skills, VIVEL co-developed a seven-session training program with a transdisciplinary panel of field experts. The sessions, held at the PCZ level, brought together diverse providers from the same zone to practice GOC through assignments and peer reflection sessions. Trainers with subject-matter expertise and experiential knowledge facilitated the sessions. Participants in this training are well-positioned to gain insights into the behavioural change required to move from problem- to goal-oriented care. This understanding is key to advancing knowledge on how GOC can be implemented in practice and may inform future workforce education and training [24]. Therefore, the aim of this study is to explore how PCPs experience the behaviour change process when applying GOC in their daily work.

## Method

### Study Aim and Design

**Figure 1:**
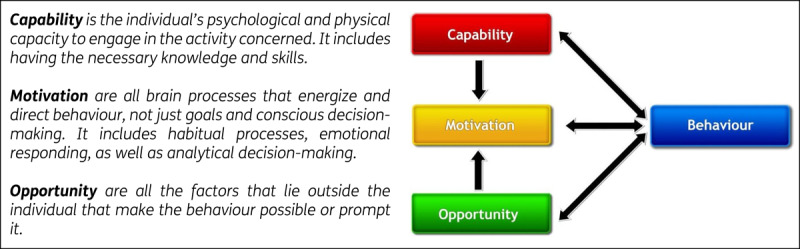
COM-B model – a framework for understanding behaviour (Michie, van Stralen & West, 2011).

This study aims to answer the research question: ‘*How do PCPs experience the behaviour change process when implementing GOC in their practice six months after participating in an interprofessional training on GOC?*’. A qualitative generic approach [25] was used as the study design. The study design combines a deductive focus on behaviour change, using the COM-B model as a theoretical framework, with an inductive approach to studying PCPs’ experiences. In this study, the COM-B model serves as the basis for the theoretical assumptions on behavioural change [26]. The COM-B model, developed by Michie and collegues (2011), considers behaviour as the dynamic interplay of three inter-related components: capability (C), opportunity (O), and motivation (M). Behaviour occurs when an individual has the capability and opportunity to engage in the behaviour and is more motivated to carry out that behaviour than others. Capability is defined as an individual’s ability to physically and psychologically perform the behaviour, while opportunity refers to external factors that enable or prompt the behaviour. Motivation encompasses basic drives, automatic processes, and reflective processes. These elements, as illustrated in Figure 1, interact to influence behaviour change or sustain a behaviour once it has been integrated into an individual’s habitual patterns of behaviour [27]. Previous research has shown the usability of the COM-B model as a theoretical framework to gain insights into the different components impacting provider behaviour change in healthcare settings [28,29]. Figure 1 presents an overview of the COM-B model, which served as the theoretical framework guiding the deductive component of the analysis.

### Sampling

A purposeful convenience sample recruited PCPs trained in GOC based on their participation in the interprofessional GOC training by VIVEL. The term *PCP* is used broadly here to include both health and social care professionals working in primary care such as pharmacists, physiotherapists and social workers. Since 2022, VIVEL trained 50 PCPs across five PCZs in Flanders. All trainees who completed the program between March 2022 and January 2023 were invited via email to participate in a study on their GOC implementation experiences.

### Data Collection

Six months post-training, focus groups were held in each PCZ where the VIVEL training took place to reduce participants’ travel. These discussions provided a space for participants to share, refine, and reflect on their experiences, making focus groups a suitable data collection method [30]. By encouraging collective sense-making, this approach aligned with the PC-IC philosophy of developing a shared vision. The focus groups captured a range of experiences, guided by the COM-B model as the theoretical framework.

A semi-structured focus group guide with five open-ended questions, revised twice for clarity and neutrality, facilitated the discussions (see Supplementary File 1). The questions explored participants’ experiences with behaviour change, including their motivation to apply GOC, supporting skills, and challenges. All focus groups were audio-recorded and transcribed verbatim.

The first author, who moderated all focus groups, had prior experience with GOC through involvement in related projects and training initiatives, which may have helped build trust with participants by providing a shared understanding of the concept. The research team included members with diverse professional backgrounds and varying levels of familiarity with GOC, which enabled critical questioning of assumptions and interpretations. This collaborative approach helped to balance the moderator’s insider perspective with external viewpoints, acknowledging that researcher influence is inherent in qualitative research.

### Data Analysis

A theoretical thematic analysis following Braun & Clarke’s method was used, supporting the deductive-inductive study design [31]. Two researchers, one junior (R.H.) and one senior (P.B.), independently coded the transcripts into the COM-B model’s components: *capability, opportunity*, and *motivation* (deductive), guided by the definitions in Figure 1 [27]. Consensus on the initial coding was reached within the team. Data within each component were then coded inductively to generate initial themes.

The analysis acknowledged that coding and theme development are inherently interpretive acts. Instead of seeking a single ‘correct’ reading, dialogue between researchers with different backgrounds generated a richer, reflexive account of the data. Team discussions refined initial codes into final themes and subthemes, ensuring consistency with the theoretical framework while maintaining reflexive openness. Supplementary File 2 illustrates this process by showing how original data evolved from theoretical coding based on the COM-B model to a sub-theme and then to an overarching theme.

## Results

### Participant characteristics

A total of 22 PCPs from five primary care zones participated in focus groups in June 2023, with three to six participants per zone. One focus group was conducted online due to participants’ preference. Participants represented various disciplines, ensuring a multidisciplinary perspective. Discussions lasted between 1 hour 51 minutes and 2 hours 20 minutes. Table 1 provides an overview of the participants.

**Table 1:** Overview of participants.


NR. PARTICIPANT	PROFESSION	WORK CONTEXT	PRIMARY CARE ZONE

1	Pedagogue^1^	Community centre for people with intellectual disabilities	A

2	Social worker	Mutual insurance company^2^	A

3	Social worker	Nursing home	A

4	Social worker	Mutual insurance company	A

5	Job coach & social work	Home care service	B

6	Social worker	Home care service	B

7	Social worker	Mutual insurance company	B

8	Pedagogue	Community centre	B

9	Social nurse	In- and outpatient centre for people with disabilities	C

10	Social worker	Social services in hospital	C

11	Pedagogue	Home care for people with ABI	C

12	Social worker	Mutual insurance company	C

13	Nurse	Home care service	C

14	Social worker	Mutual insurance company	D

15	Social worker	Mutual insurance company	D

16	Social worker	Public social welfare centre	D

17	Dietician	Independent practice	E

18	Psychologist^3^	Independent practice	E

19	Physiotherapist	Independent practice	E

20	Nurse	General practitioners’ practice	E

21	Social worker	Mutual insurance company	E

22	Pedagogue	Mental health outreach team	E


^1^“Pedagogue” refers here to a professional supporting adults with intellectual disabilities. ^2^“Mutual insurance companies” are non-profit health insurance funds with responsibilities in preventive health and care coordination. ^3^Independent practice psychologists in Belgium are accessible without referral and are considered part of primary care.

In all focus groups, participants indicated a pre-existing interest and affinity for GOC, as it was already present in their work, training, or personal values. However, they expressed a need for further guidance on its practical implementation in daily practice, which remained unclear to them.

**Figure 2:**
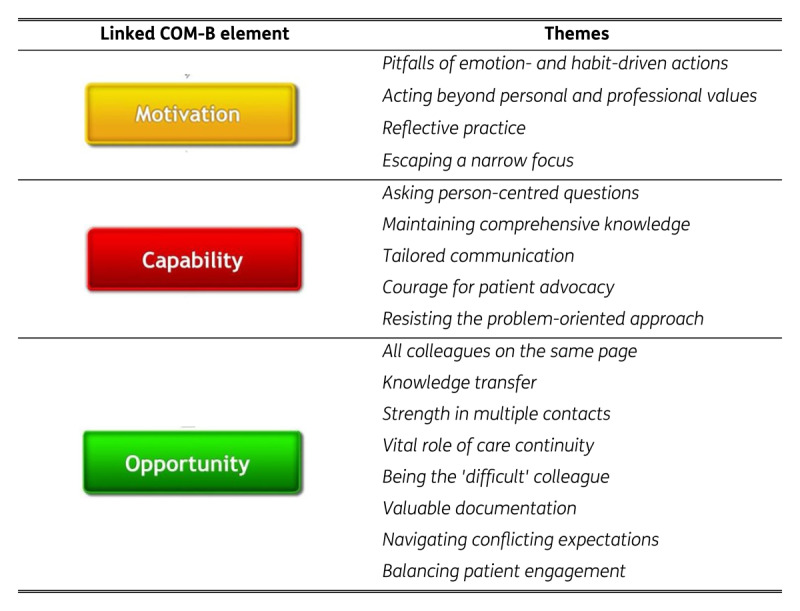
Overview of the themes.

The themes are discussed in order on what participants emphasized in the results. They indicated the themes linked to the *motivation* component as the fundamental drivers for implementing GOC in practice. These themes will be followed by those related to *capability* and *opportunity*. Figure 2 provides an overview of the themes identified in this study presented within the COM-B model.

### Motivation

Participants emphasized the importance of developing personal and professional awareness of their behaviour. This awareness helps them recognize how routines and emotions shape their actions, allowing for reflection on the connection between their actions, emotions, and values. Through this reflective process, they more consciously consider the relevance and meaning of their interventions, enabling them to align their behaviour with GOC principles.

Pitfalls of emotion- and habit-driven actions: The participants expressed a deeper understanding of how routines, habits, and emotions influence the behaviour changes required for implementing GOC. They recognized that embracing GOC often requires breaking away from established practices, processes and routines. Some participants noted that, they sometimes struggle to move away from a problem- or provider-driven approach. They highlighted the challenge of changing work routines, especially when quick decisions are needed. In urgent situations, they experienced that they tend to act more problem-oriented than goal-oriented. They described that it takes time to step back from initial emotional responses to reflect on a care situation. They felt that acting out of routine hinders opportunities for reflection. Finally, some participants expressed fear of deviating from their problem-oriented routines, worrying that it might lead to professional negligence toward their patients.

*“…There may be safety risks, but you can’t just impose that. Like ‘You have to do this.’ Instead, you should start the conversation though, like ‘There are a few things I’m concerned about.’.”* (P11, PCZ C, Pedagogue)

Acting beyond personal and professional values: Participants found that providing GOC challenged them to align actions with patient preferences. In this process of change they realized that some actions were mainly driven by their own values which might not necessarily comply with those of the patient. This awareness extended to both their professional and personal values, with values being understood as the meaningful beliefs that motivate professionals in their actions. They stressed the importance of reflecting on whether care actions align with the patient values and expectations. This led them to realize that patients’ preferences, rooted in patients’ values, might conflict with their personal or professional values. Participants emphasized the need to manage feelings of powerlessness and rise above these conflicts to deliver GOC.

*“Because I knew that patient doesn’t want that [going to a day care centre]. And I really had mixed feelings like ‘okay, if I really want to work goal-oriented, I’m not going to force it. So, we had a conversation.”* (P9, PCZ C, Nurse)

Reflective practice: Participants shared that providing GOC prompted them to reflect more frequently on their professional actions and on the added value of their actions taking the patient’s preferences into account. This introspection led them to question the broader purpose of their role as a care provider, motivating them to seek innovative approaches to adapt their professional actions according to patients’ personal goals.

Escaping a narrow focus: When participants or their teams used a GOC approach in a patient’s situation this often provided them with new insights. They experienced that GOC helped them to broaden their perspective on the situation. Especially in ‘complex cases’, they felt at greater risk of acting from emotion- or habit driven routines, which causes them to lose sight of the bigger picture and the patient’s personal goals. In these complex cases, participants noted that GOC helped them to consciously reassess the relevance of specific care actions by clarifying the patient’s wishes and personal goals.

*“It was such a tangled situation, that family history, all the stories from the children… That I, got so sucked into it… And I needed to get some distance… to sort of open it up again… Like ‘Wait, what was your wish again?’”* (P7, PCZ B, Social worker)

### Capability

When understanding the skills and knowledge participants use to initiate behaviour change towards GOC, participants rely on the reflective process outlined in the motivational component. They consciously slow down their thinking and extend care organization to avoid a problem-based approach. Taking time to understand patients’ interests and motivations helps them better advocate for patients’ wishes in conversations with colleagues.

Asking person-centred questions: The participants indicated the application of GOC in their communication style by asking broad questions, such as asking patients about their interests and expectations. Additionally, they more frequently probed into the underlying meaning behind expectations or wishes expressed by patients. Asking ‘why’ something was important to the patient was an important way for them to gain insight into the patient’s motivation.

*“I think I’ve now become a nurse who listens much more than I used to. You can come to me because you’re in pain, but then I think, okay, and what else? I still don’t really know anything about you. Do you stay at home, or do you work, do you have children, what keeps you busy, and how do you see the future? I used to be much less concerned with those things.”* (P20, PCZ E, Nurse)

Maintaining comprehensive knowledge: Participants emphasized that having a broad knowledge of the various available services is supportive when addressing patients’ goals. Keeping this knowledge up to date was not easy given the rapid changes occurring in the service landscape.

Tailored communication: When engaging with patients and caregivers, participants reflected that they consciously paid attention to their body language and choice of words to establish connection and trust in the interaction. They indicated that they communicated their message tailored to the patient to maximize the chances of good understanding. Examples included adapting language to the patient’s dialect, visualizing information schematically or choosing a deliberate physical position at the table with the patient. Additionally, consideration was given to who is best positioned in the team to deliver a message, with the trust relationship primarily used as a criterion for making this choice.

*“You must be a bit of a chameleon…to be able to change colours depending on who sits in front of you. Sometimes that means speaking nice Dutch or chatting more casually (adjusts accent) … sometimes you change a bit who you are as a person, without losing yourself.”* (P1, PCZ A, Pedagogue)

Courage for patient advocacy: Participants noted that from the perspective of GOC, they felt more courageous to address meaningful matters for their patients during conversations. Particularly in discussions with colleagues, they dared to bring up meaningful issues for the patient, even if it meant engaging in debate with colleagues. Leveraging their extensive patient knowledge, they felt empowered to challenge colleagues on proposals deemed unfeasible or not aligned with patients’ goals. Participants acknowledged that this demanded assertiveness. They found that discussing concerns or considerations regarding the patient’s goals, with colleagues, caregivers, or the patients themselves, fostered a shared understanding among all parties. According to them, this facilitated the progress in the care trajectory.

*“Or when I notice in a meeting that something else is important, something we must not lose sight of. I think you also have to dare to change your style a bit. During patient meetings. You can sit there as a silent voice, but you can also dare to speak up and advocate for it.”* (P10, PCZ C, Social worker)

Resisting the problem-oriented approach: Participants indicated that within GOC, they employed strategies to avoid reverting to a problem-oriented approach. They deliberately slowed down the process of organizing care, allocating more time for patients to consider information and proposed actions. This involved scheduling multiple appointments and encouraging reflection between consultations. Participants acknowledged that this way of organizing their work differed from their previous practice, during which they may have pressured patients more to adhere to certain treatments or advice.

### Opportunity

Among factors that lie outside the participants impacting their behaviour change towards GOC, mainly social factors seem to have an impact. Support from colleagues and supervisors influences their empowerment to provide GOC. Participants fear damaging professional relationships if they pursue GOC when colleagues do not acknowledge its relevance. Additionally, organizational factors like long-term patient follow-up and the documentation of patient goals in health record system are seen to facilitate GOC implementation.

All colleagues on the same page: Participants emphasized the value of support from their supervisors. Most participants work in organizations with supervisors and felt supported and encouraged by them to explore ways to implement GOC in their work. All participants agreed that for optimal implementation of GOC, it is essential to involve all colleagues in this care approach. The support that some participants already experienced from their supervisors was seen as a prerequisite to bring the whole team on board.

*“I think everyone should be included, because if your administrative staff isn’t on the same page with the idea of GOC and gets those calls from patients and makes appointments, then they won’t know how… No, everyone must be on board.”* (P16, PCZ D, Social worker)

Knowledge transfer: The primary strategy participants use to engage all colleagues is education. Several participants have spontaneously given presentations or shared knowledge about GOC with their colleagues.

Strength in multiple contacts: Participants acknowledged that while the initial perception of GOC as time-intensive posed a challenge to feasibility, their perspective shifted as they gained more experience. Rather than viewing time allocation as the main barrier, they emphasized the importance of continuity of care and maintaining ongoing, meaningful contact with patients. They highlighted the value of reflecting on the patient’s care journey at various touchpoints, contrasting with the view of colleagues who resisted GOC due to time constraints.

*“At the beginning of the training, when we were asked what we needed, I said ‘time.’ But I don’t even consider time as an issue anymore. I try to manage within our time constraints. Now, I just need to be able to do it, to have enough contact with the same client. That’s frustrating for me. Yes, that continuity.”* (P12, PCZ C, Social worker)

Vital role of care continuity: Participants discussed changes in government healthcare policy over the past few years, which have resulted in shorter and fewer patient contacts, posing a challenge to providing GOC. Continuity in the patient-provider relationship was deemed essential for GOC. Participants found that GOC lacks effectiveness when there is only brief, one-time contact with a patient and no further interaction. They advocated for multiple or recurring contacts with patients, either directly or facilitated by colleagues, to enable effective GOC.

Being the ‘difficult’ colleague: Participants experienced a sense of tension when engaging with partnering organizations or colleagues who operated from a different mindset or approach. In interprofessional meetings, when highlighting patient goals, they felt often as the ones asking ‘difficult questions’. While they viewed this as a crucial aspect of GOC, it led to feelings of discomfort and intimidation. Participants expressed concerns that emphasizing patient goals could strain professional relationships and potentially jeopardize collaboration with colleagues who might prioritize other aspects of care.

Valuable documentation: Participants expressed challenges in integrating GOC into their practice through proper documentation in digital health records. While both policy and supervisors emphasized the importance of recording GOC-related information, participants felt that the available systems were inadequate for capturing the nuanced and meaningful aspects of patient care. They noted that current documentation tools often overlook personal, valuable information that could benefit alignment with colleagues in providing GOC. As a result, much of the critical information remains undocumented, residing only in the minds of the practitioners.

*“You can still make your report, but there’s a lot more in your head than what you put in the medical file, you know.”* (P12, PCZ C, Social worker)

Navigating conflicting expectations: Participants encountered challenges in providing GOC when informal caregivers had differing expectations or needs from those of the patient. In these cases, participants prioritized the patient’s personal goals, but found their ability to act constrained by the conflicting perspectives of caregivers.

Balancing patient engagement: Participants increasingly sought to involve both patients and their caregivers in healthcare decisions, noting that this shift aligns with a trend of patients advocating for themselves. However, they identified a pitfall in assuming that all patients are able or willing to express their goals. Some patients still prefer to rely on professionals or caregivers to decide for them. Similarly, caregivers accustomed to making decisions for the patient, struggled with the shift to a more patient-centred approach, where professionals involved the patient in the decision-making process. This dynamic required balancing effective involvement without overestimating the readiness of patients or caregivers for full participation.

## Discussion

This study explored the behaviour change process among PCPs implementing GOC in daily practice, showing that reflective practice emerged as a key driver of this process. Reflectivity helped participants recognize how their values, routines, and emotions shape their actions, while capabilities such as asking person-centred questions, expanding knowledge, and advocating for patients supported this change. Opportunities for change were influenced by the need for colleagues alignment on GOC, and support from supervisors and teams. Addressing documentation challenges was found to be crucial for ensuring continuity of care. These findings add new insight by demonstrating how behaviour change unfolds dynamically across the COM-B components, with reflective practice acting as a crucial catalyst.

Although participants identified a set of skills they associate with providing GOC, the value of these skills only seems to emerge when professionals also have the appropriate motivation to demonstrate them. This finding highlights a clear connection between the components of *capability* and *motivation*. Participants described communication skills—such as asking person-centred questions and being conscious in their way of communicating—as helpful when providing GOC. However, previous research involving general practitioners (GPs) showed that simply asking about patients’ quality of life (QOL) did not automatically lead to GOC. Instead, GPs tended to medicalize QOL information rather than addressing patients’ wishes and preferences [14]. This illustrates that behavioural change towards GOC requires more than acquiring communication techniques; it calls for a mindset that enables providers to align care with patients’ goals. Literature suggests that effective GOC implementation depends on developing adaptive leadership among healthcare providers (HCPs). Adaptive leadership enables providers to distinguish between technical problems—well-defined issues with clear solutions—and adaptive challenges, which are complex, ambiguous, and without straightforward answers. Unlike technical problems, adaptive challenges are often rooted in values, beliefs, and loyalties, requiring providers to change established practices, priorities, and relationships [32]. In this context, adaptive leadership goes beyond asking, “*What matters to you?*” as a technical question it involves aligning care with patients’ wishes and needs, while managing uncertainty, fostering equal partnerships, and being open to change. A PCP with adaptive leadership skills engages in conversations based on equal partnership, moving beyond a conventional command-and-control approach [33].

Reflective practice emerged as a key strategy supporting participants in addressing patients’ personal goals. By reflecting on the interplay of care actions, emotions, and personal values, providing GOC becomes a conscious cognitive process. A systematic review on reflexivity describes it as reflecting on one’s identity, beliefs, and values [34], closely aligning with participants’ descriptions of their reflective practices. This review also confirms participants’ experiences that reflexivity can influence attitudes and behaviours of HCPs. Research further links reflection skills in HCPs to fostering equitable patient relationships [35] and reducing paternalistic approaches in care [36]. A person’s ability to be reflective is driven by motivation but can be significantly shaped by the environmental context in which HCPs operate [37]. According to the COM-B model and other behavioural frameworks, motivation comprises two subsystems: the automatic system, driven by emotions and habits, and the reflective system, which prompts deliberate thought and self-reflection [27]. This final system is primarily activated in learning contexts or complex situations [38]. Participants in this study, who voluntarily attended GOC training, likely already possessed an interest in applying GOC, which enhanced their motivation to learn [39]. Their experience that reflection acted as an important strategy for applying GOC in their practice suggests they perceive their work environment as a learning context. This aligns with the concept of Workplace Learning (WPL), where “learning occurs at work, through work, and for work” [40]. For WPL to succeed, a supportive environment is essential. Resources like electronic patient records or workspace design can facilitate learning by strengthening connections between actors working towards shared goals [41]. In this study, themes within the *opportunity* component highlight contextual factors shaping behavioural change toward GOC. Participants noted, for instance, how organizational capacity for long-term patient follow-up and shared documentation systems can act as enabling elements.

Integrating GOC into professional behaviour requires a time investment in two distinct ways. First, discussing patients’ personal goals takes time. Building trust and exploring interests and preferences of the patient are recognized as necessary time investments in providing GOC [16,42,43]. Second, participants consciously allocated time within the care trajectory to reflect on the care plan with patients. Research confirms that time is a prerequisite for reflection [39]. However, when HCPs perceive their work environment as time-pressured, behaviour often reverts to habits and emotions rather than reflective practice [37,44]. Participants in this study confirmed these challenges, which is unsurprising given the resource and staff shortages in Flemish primary care [45]. Yet, despite these constraints, they deliberately made space for reflection and considered it a crucial enabler of behavioural change towards GOC. This finding suggests that, contrary to the commonly reported ‘no-time’ barrier in other studies [14], participants found value in prioritising reflection. Future research should investigate what supports reflective practice among HCPs, even in time-constrained environments.

Beyond tangible resources, social contextual factors appear in our study to play a significant role in driving behavioural change towards GOC. Themes within the *opportunity* component illustrate how interactions with colleagues, supervisors, patients, and caregivers shape the adoption of GOC. Participants emphasized the importance of team alignment in prioritizing patient goals, yet some colleagues maintained a problem-oriented approach. This aligns with Dutch primary care research, where teams trained in person-centred care reported increased focus on patient goals in interprofessional meetings, though meeting observations did not confirm this [46]. Similar discrepancies in perceptions between provider teams and patients regarding GOC have been observed in both Flemish [15] and Swedish [47] primary care contexts. This difference in perception between teams and patients reveals a gap for further research.

Participants in this study often found themselves advocating for patient goals during interprofessional meetings, as colleagues did not consistently operate from a GOC approach. This advocacy required courage, as participants risked being perceived as the ‘difficult’ colleague—a dynamic closely tied to moral distress. Moral distress arises when HCPs feel obstructed from acting in alignment with their moral beliefs due to internal or external constraints [48]. In teams, insufficient collaboration or communication can lead to care misaligned with patients’ goals, creating moral distress. Similarly, conflicts between patients’ preferences and providers’ perceptions of what is important for their care can contribute to feelings of powerlessness. Additionally, the pitfall of assuming that every patient can or wants to express their personal goals can also contribute to moral distress [47,48]. To address these challenges, creating a workplace culture that encourages discussion and reflection is essential [34]. Protected time for team discussions, combined with opportunities for individual reflection, can enhance collaboration, reduce moral distress and increases job satisfaction [39,48,49].

This study underscores that behavioural change in GOC is strongly shaped by context. Reflective skills were central to how participants adapted their behaviour, highlighting the need for supportive work environments. As GOC skills align with integrated care competencies—such as building a shared vision, sharing accountability, and organizing interprofessional networks—future research could explore whether integrated care settings provide supportive contexts for developing and sustaining GOC behaviours [39,50]. The importance of both functional and normative integration for GOC implementation was also emphasized [51]. Training clinicians, managers, and patients in GOC may further support the implementation of integrated care models [7]. Finally, as reflective practice appears central to adaptive leadership in GOC, identifying other adaptive skills will help refine the required skillset. Without such efforts, GOC risks being reduced to a superficial question—“*What matters to you?*”—leaving teams uncertain about how to act on this meaningful information [32].

## Lessons Learned

- **Prioritize Reflective Practice:** Embedding reflective practice helps PCPs and teams align care with patients’ goals, even when existing professional values and workflows create resistance.
- **Address Resource Constraints:** Time and staffing constraints may limit reflection, but making it a core habit is essential for behavioural change toward GOC.
- **Allocate Time for Reflection:** Reflexivity requires protected time—not only with patients but also within teams—to consider how and which goals shape care decisions.
- **Foster Team Alignment:** Strengthening reflective skills across the team fosters shared understanding and consistency in pursuing patients’ personal goals.

## Strenghts and Limitations

The study sample consisted of PCPs who had completed GOC training, reflecting a pre-existing interest in the concept. This may have influenced the findings, as behaviour change processes could differ for providers with less motivation or interest. Recruiting trained participants was intentional to ensure that the behaviour change process was studied from a shared understanding of GOC. The sample included various healthcare disciplines in primary care, enhancing transferability of the findings. However, the absence of general practitioners (GPs) is a limitation. Although they were invited, GP enrolment in the training was already low (only one per PCZ), likely due to its intensive, multi-session format scheduled during working hours. These conditions reduced the likelihood of their participation in the daytime focus groups, which also offered no compensation [52,53]. While GPs play a central role in chronic care management, the design of both the training and this study reflects the interprofessional context in which GOC is often embedded. The findings therefore remain relevant, as GOC is a cross-disciplinary approach [7]. Future research should aim to include all primary care disciplines, such as GPs, pharmacists, and psychologists by offering more flexible data collection times to accommodate schedules [54].

The choice of focus groups as a data collection method is a strength of this study. Participants were familiar with one another through the training, likely fostering trust and open discussion. This pre-existing relationship may also have increased participation by creating a sense of reunion among the PCPs [55].

Importantly, the study sought to capture participants’ reflections on the behaviour change process in GOC rather than their actual observed behaviour. There is a risk that participants may have overestimated or underestimated their behaviours, as certain supportive actions toward GOC may go unnoticed by the providers themselves and thus were not discussed. Qualitative studies using observational methods are needed to capture the actual behaviour of PCPs when implementing GOC [56]. A key strength of this study is the diverse expertise of the research team, which included implementation scientists, behavioural scientists, GOC, interprofessional collaboration, and primary care. This collaborative approach strengthened the study’s credibility and helped mitigate potential biases in data interpretation.

## Conclusion

This exploratory study identified key aspects of the behaviour change process that primary care providers experience when implementing goal-oriented care in practice. Motivational factors, particularly reflective practice, emerged as key drivers of behaviour change, helping participants recognize how their values, routines, and emotions shape their actions. Participants identified important skills, such as asking person-centred questions, expanding their knowledge, and strengthening their advocacy for patients. Furthermore, opportunities for change were influenced by the need for alignment among colleagues regarding the GOC approach, as well as the support of supervisors and teams. Addressing documentation challenges will also be vital in facilitating continuity of care.

## Additional Files

The additional files for this article can be found as follows:

10.5334/ijic.9067.s1 Supplementary File 1. Focus group guide_ GOC Behavioural change in practice _ENG version.

10.5334/ijic.9067.s2 Supplementary File 2. Example of theoretical thematic analysis.
