# Supplementary material for: Behavioural Change in Practice: Primary Care Providers’ Journey Towards Goal-Oriented Care
Source: Int J Integr Care. 2025 Dec 19;25(4):22. doi: 10.5334/ijic.9067 (PMC12716247; doi:10.5334/ijic.9067)
Supplement: Supplementary File 1. — Focus group guide_ GOC Behavioural change in practice _ENG version. [file ijic-25-4-9067-s1.pdf]

**Focus group guide_ GOC Behavioural change in practice _ENG version**

*The main questions guiding the focus groups are shown in* ***bold****. The questions in italics are optional, depending on the flow of the discussion.*

1. Everyone started the training from a different work context with different starting points or motivations on GOC. To better understand those starting points, I like to ask first: **What brought you to this training on GOC? What motivated you to attend this training on GOC?**

2. **Since attending the training on GOC, what has changed in your work or work environment?**
    a. *How is this change related to GOC for you?*
    b. *What have you started doing differently/extra to make this change happen?*
    c. *In what ways are you currently applying GOC in your work context?*
    d. *What did you learn from the training that contributed to this?*
    e. *What expectations did you have prior to the training about what you would learn? (any expectations from the environment?)*

3. **In your opinion, how does GOC manifest itself in daily practice? In mindset, behaviour, or both?**
    a. *How can you recognize someone practicing GOC?*

4. **Can you tell me something about the challenges you experienced in applying what you learned in the training on GOC to your work?**
    a. *What kind of support would help you address these challenges?*
    b. *What would you still like to change in your work regarding GOC and do you feel skilled enough to do so?*
    c. *What expectations were not met? And how could these still be met through training?*

5. **What skills or competencies do you believe a primary care provider needs in order to apply GOC in his/her work?**
