# Supplementary material for: Behavioural Change in Practice: Primary Care Providers’ Journey Towards Goal-Oriented Care
Source: Int J Integr Care. 2025 Dec 19;25(4):22. doi: 10.5334/ijic.9067 (PMC12716247; doi:10.5334/ijic.9067)
Supplement: Supplementary File 2. — Example of theoretical thematic analysis. [file ijic-25-4-9067-s2.pdf]

Supplementary File 2 Example of theoretical thematic analysis

<table>
<tr><th>Focus group extract</th><th>Theory-coding according to COM-B</th><th>Sub-theme</th><th>Theme</th></tr>
<tr><td rowspan="6">[transcript focus group PCZ C]
…
Participant 12: I think that alertness…. just being aware of it. Yes. Paying more attention to things. You can especially see this during a home visit. From photos, souvenirs.[A] And not just thinking, ‘I’m seeing this, but I don't have time for it, so I won't ask about it.’ Sometimes that's really bad... [B] You just must address it once. Yes. Bring it up at some point. Yes.

Participant 9: Indeed, when you see pictures...being like ‘Ah, this is someone who values family. A family man, family woman.’ Yes. Or you see photos of holidays, traveling, something…. Those are things that you then, that you don't describe in a report to your colleague.[C]

Participant 12: No. Or that you might say, ‘You've travelled so much, don’t you miss it now?’[D] Because people often say, that it’s not possible anymore for them. But it could be. There are adapted vacations.[E] And it’s not that I didn’t do that before, but I think I’m more aware of it now…Yes.[F]
…</td><td>[A]: In the data up until A, participant 12 talks about how her awareness and attention can contribute to goal-oriented care. Therefore, this data is coded as MOTIVATION.</td><td>Routine takes over</td><td>Escaping a narrow focus</td></tr>
<tr><td>[B]: In the data up until B, participant 12 talks about how she tries not to use a lack of time as an argument to avoid asking the patient questions. Therefore, this data was coded as OPPORTUNITY.</td><td>Time constraint resistance</td><td>Vital role of care continuity</td></tr>
<tr><td>[C]: In the data up until C, participant 9 talks about how valuable personal information about a patient is sometimes difficult to report with colleagues. Therefore, this data was coded as OPPORTUNITY.</td><td>Documenting GOC</td><td>Valuable registration</td></tr>
<tr><td>[D]: In the data up until D, participant 12 describes how, from his presence in a patient's home environment and triggers in the house, he will ask personal questions to the patient. Therefore, this data was coded as CAPABILITY.</td><td>Broad questioning</td><td>Asking person-centred questions</td></tr>
<tr><td>[E]: In the data up until E, participant 12 describes how, based on his knowledge about traveling with a disability, he can inform patients who are convinced that it is no longer an option for them. Therefore, this data was coded as CAPABILITY.</td><td>Broad knowledge on services</td><td>Maintaining comprehensive knowledge</td></tr>
<tr><td>[F]: In the data up until F, participant 12 describes how he has become more aware of the skills he uses to inform patients. Therefore, this data was coded as MOTIVATION.</td><td>Reflecting on relevance</td><td>Reflective practice</td></tr>
</table>
